# 3D seismic modeling of the Amal oil field to evaluate CO_2_ storage potential in depleted reservoirs, Southern Gulf of Suez

**DOI:** 10.1038/s41598-025-03032-5

**Published:** 2025-05-26

**Authors:** Mohammed Amer, Walid M. Mabrouk, Amr M. Eid, Ahmed Metwally

**Affiliations:** Geophysics Department, Faculty of Science, Cairo University, Giza, 12613 Egypt

**Keywords:** Carbon capture and storage, 3D Geological modeling, Seismic interpretation, Sequential gaussian simulation (SGS), Upper Rudies, Gulf of Suez, Geology, Geophysics, Sedimentology, Tectonics

## Abstract

The Amal Oil Field in the Southern Gulf of Suez presents significant potential for Carbon Capture and Storage (CCS). This study integrates 3D geological modeling, seismic interpretation, and petrophysical analysis to assess the field’s suitability for CO_2_ sequestration. The structural analysis identifies a primary horst block bounded by major normal faults, providing an effective structural trap for CO_2_ storage. Stratigraphic studies confirm the presence of robust sealing formations, including the Kareem shale and the evaporite-dominated Zeit and South Gharib Formations, ensuring long-term containment. Petrophysical evaluation of the Upper Rudies reservoir reveals favorable conditions for CO_2_ injection, characterized by low shale volume, moderately high effective porosity, low water saturation, and adequate permeability. Reservoir property modeling, conducted using sequential Gaussian simulation (SGS), a statistical method used to distribute reservoir properties, such as porosity and permeability, throughout the reservoir by generating multiple possible scenarios based on a Gaussian distribution model, demonstrates significant lateral and vertical heterogeneity, with the central horst block exhibiting the highest storage potential. Permeability distribution varies from 0.1 to 100 mD, with an average of 10 mD in key reservoir zones, further supporting its suitability for CO_2_ injection. CO_2_ storage capacity estimation, incorporating grid pore volumes, CO_2_ density, formation volume factor, and storage efficiency coefficient, suggests a storage potential ranging from 3.6 to 48.5 million tons. Spatial analysis highlights the central and northwestern regions as the most promising areas for injection due to higher porosity and net pay thickness. The Gulf of Suez boasts a unique geological setting, providing excellent structural traps for hydrocarbon and CO_2_ storage. Its well-developed infrastructure, including extensive pipelines, processing facilities, and existing wells, supports efficient CO_2_ transportation and injection, enhancing the feasibility of large-scale CO_2_ storage with minimal additional investment. The region’s strategic location also enhances its role in global trade and energy logistics. This study provides a comprehensive workflow for evaluating depleted hydrocarbon reservoirs for CCS applications, offering valuable insights for future CO_2_ sequestration projects in the Gulf of Suez, a region underexplored in CCS literature. The findings contribute to Egypt’s national carbon reduction initiatives and support global climate mitigation strategies.

## Introduction

As a key player in the global energy market and a country rich in natural resources, Egypt faces the pressing challenge of achieving sustainable development while addressing its carbon footprint. Carbon Capture and Storage (CCS) is emerging as a cornerstone of Egypt’s strategy to combat climate change and transition toward sustainability. With an economy heavily reliant on fossil fuels, integrating CCS technology can significantly reduce carbon dioxide (CO_2_) emissions and support global decarbonization goals. The Southern Gulf of Suez, a region of immense geological and energy significance, offers promising opportunities for implementing CCS, leveraging its extensive oil and gas reservoirs to store atmospheric CO_2_ securely^1,2^. This aligns with Egypt’s commitment to sustainable resource management and environmental stewardship.

Globally, CCS is gaining momentum as an essential tool to curb greenhouse gas emissions. As nations strive to meet ambitious carbon neutrality targets by mid-century, the deployment of CCS technologies is becoming integral to mitigating the environmental impact of the industrial and energy sectors^60–65^. Geological CO_2_ sequestration has shown remarkable potential for long-term carbon storage. Depleted oil and gas reservoirs, deep saline aquifers, and unmineable coal seams are recognized as viable storage sites due to their geological characteristics, storage capacity, and pre-existing infrastructure^3^. Among these options, depleted oil and gas reservoirs stand out for their enhanced operability, extensive storage potential, and established sealing mechanisms, making them a prime focus for CCS efforts worldwide^4–6^.

Three-dimensional (3D) geological modeling plays a critical role in the effective evaluation of CO_2_ storage potential. By integrating well logs, seismic reflection data, and core analyses, 3D modeling enables the precise characterization of subsurface structures and reservoir properties^7–15^. This technology provides crucial insights into the structural, petrophysical, and geochemical attributes of reservoirs, enhancing the assessment of their suitability for CO_2_ injection and containment. Moreover, 3D modeling incorporates advanced simulation techniques, such as sequential Gaussian simulation (SGS), to account for reservoir heterogeneity and uncertainty^16^. These models are indispensable for optimizing site selection, estimating storage capacity, and minimizing risks associated with CO_2_ migration and leakage^17^.

While several global studies have demonstrated the efficacy of CCS in reducing carbon emissions, many challenges remain, particularly in evaluating storage capacity and understanding CO_2_ trapping mechanisms. Research has highlighted the need to investigate the interplay of geological and engineering factors that influence CCS success^2^. The Amal Oil Field, like many other hydrocarbon-rich regions, offers an excellent opportunity to implement CCS by repurposing its depleted reservoirs. The application of 3D geological modeling to assess these reservoirs is essential for ensuring their suitability for long-term CO_2_ storage while addressing concerns such as reservoir sealing and structural integrity^18^.

Egypt’s sedimentary basins, including those in the Gulf of Suez, present a unique opportunity for advancing CCS. These basins have a rich history of hydrocarbon exploration and production, making them well-studied and equipped with the necessary infrastructure for CO_2_ sequestration projects^5,19,20^. Limited studies on CCS potential in the Gulf of Suez’s depleted reservoirs, despite its mature infrastructure, highlight the need for further research. Leveraging this existing knowledge and infrastructure can significantly reduce the costs and risks associated with CCS deployment. The integration of geological, geophysical, and geoengineering data into comprehensive 3D static models can provide a deeper understanding of the region’s storage potential and support informed decision-making for sustainable energy practices^21^.

The Gulf of Suez features a distinctive geological setting characterized by a complex graben system, which offers excellent structural traps for both hydrocarbon and CO_2_ storage. This unique geological framework is complemented by the region’s extensive infrastructure, including pipelines, processing facilities, and existing wells, which facilitates efficient CO_2_ transportation and injection. These infrastructural assets significantly enhance the feasibility of large-scale CO_2_ storage with minimal additional investment. Furthermore, the Gulf of Suez’s strategic location not only boosts its role in global trade and energy logistics but also positions it as a critical hub for carbon capture and storage initiatives. The combination of geological suitability and infrastructural readiness makes the Gulf of Suez an ideal candidate for sustainable CO_2_ sequestration projects, contributing to both regional and global climate mitigation efforts.

This study focuses on the Amal Oil Field, situated in the Southern Gulf of Suez, Egypt. The field is known for its complex geological formations, especially within the Miocene sedimentary sequences, which feature the Belayim Formation, Kareem Formation, and Rudies Formation. Significant emphasis is placed on studying the Upper Rudies Formation. These formations have historically served as significant hydrocarbon reservoirs but now hold promise as candidates for CO_2_ storage. The primary objective of this research is to develop a detailed 3D static model of the Amal Oil Field to evaluate its CO_2_ storage potential. By integrating high-resolution seismic data and advanced modeling techniques, this study aims to address challenges such as reservoir connectivity, geometry, and sealing efficiency. This research will contribute to the broader goal of establishing CCS as a viable solution for reducing Egypt’s carbon emissions and fostering a sustainable energy future^22,23^.

## Geological setting

**Fig. 1:**
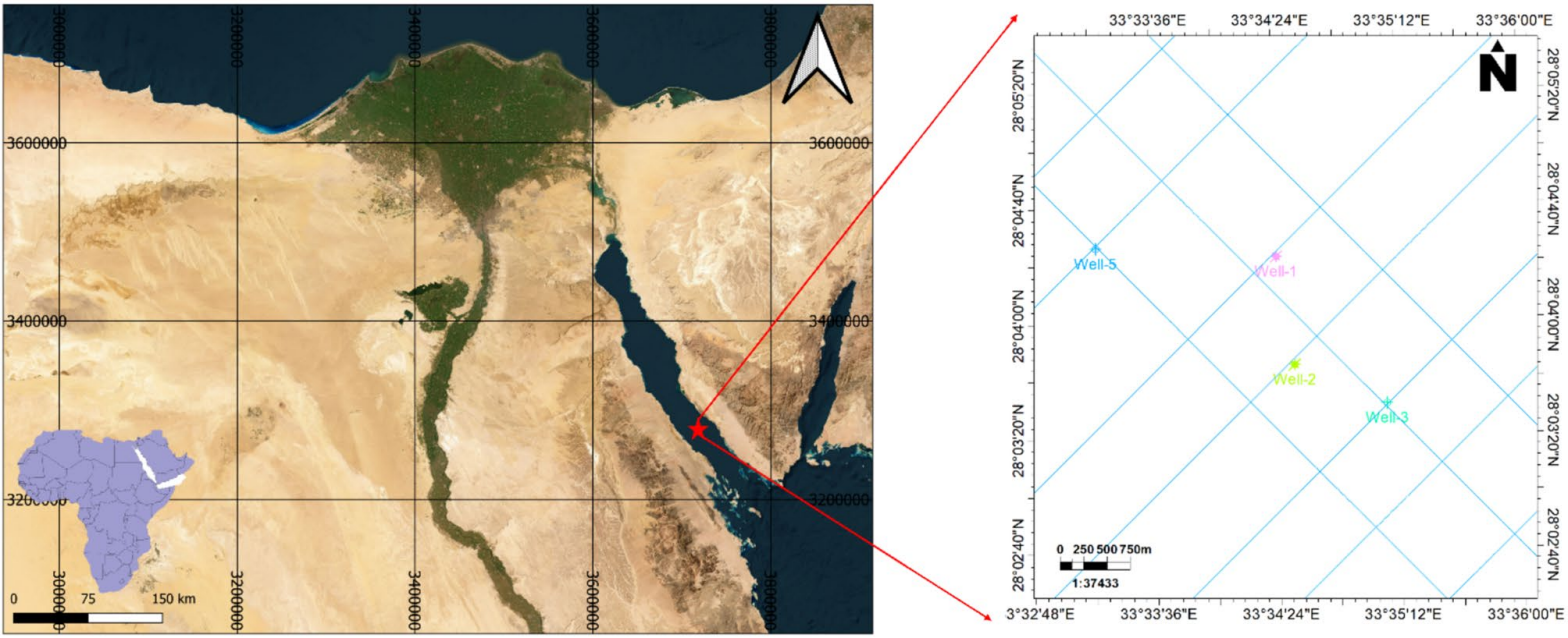
Location map of the study area illustrating seismic data coverage and the distribution of wells utilized in the Amal Field^66^.

The Amal oil field is located in the southern sector of the Gulf of Suez, offshore Egypt (Fig. 1). It lies on the western margin of the Gulf and represents an important hydrocarbon province due to its prolific petroleum system and significant production potential^24^. The field is situated within a structurally complex region that has undergone multiple tectonic events, leading to the formation of several reservoirs with varying properties. It primarily produces hydrocarbons from the Miocene-aged Rudies, Kareem, and Belayim formations, with deeper reservoirs also contributing to its commercial viability^25^. The Amal field plays a crucial role in Egypt’s petroleum industry and has the potential for further exploration and enhanced recovery techniques.

**Fig. 2:**
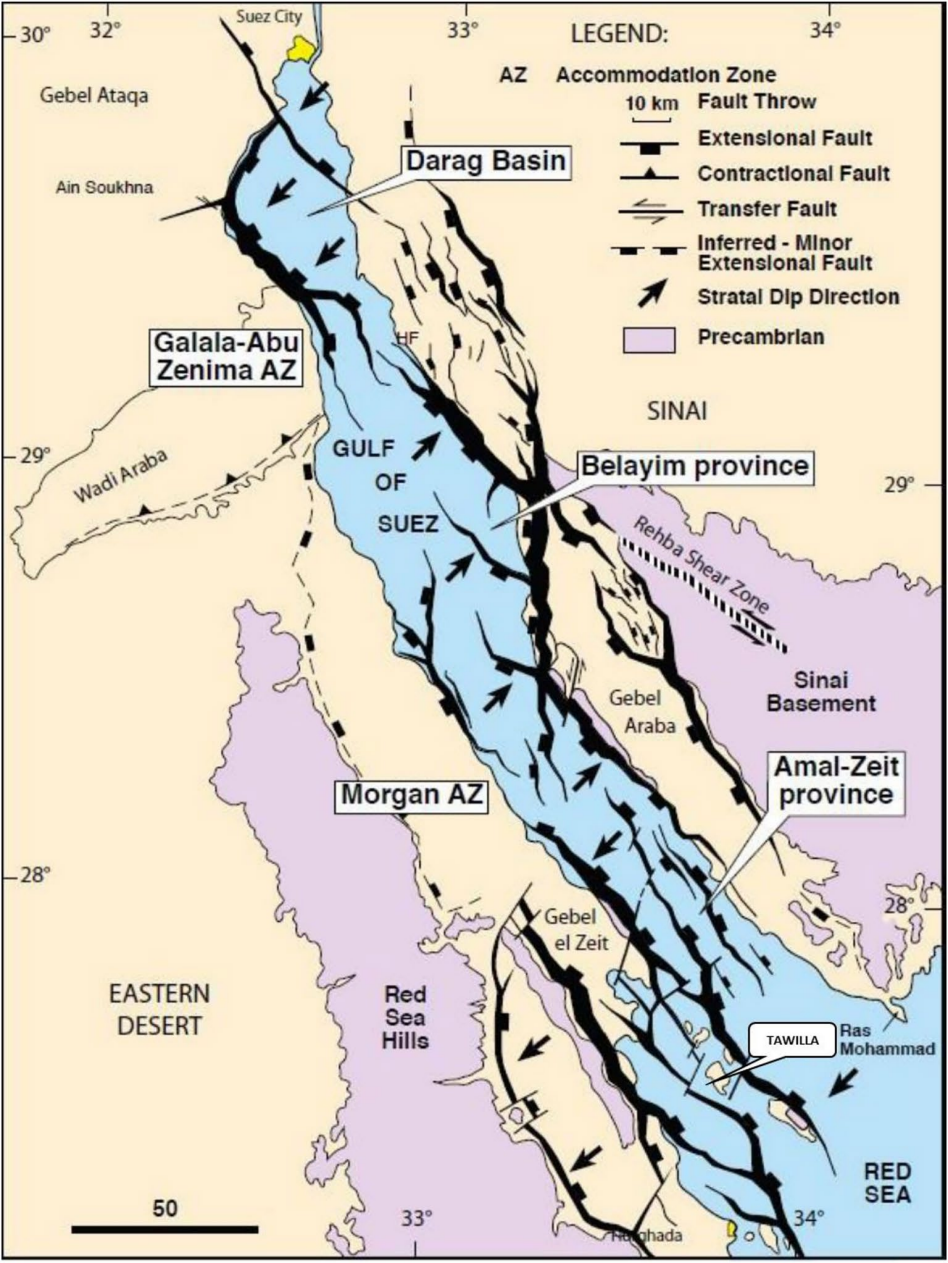
Tectonic and structural map of the Gulf of Suez, highlighting its structural trap style, adapted from^32,40^.

Tectonically, the Amal oil field is part of the Gulf of Suez rift system (Fig. 2), which formed as a result of extensional tectonics during the Late Oligocene to Early Miocene^26^. The rifting event was driven by the opening of the Red Sea, causing the stretching and thinning of the continental crust. The field is bounded by a series of normal faults that are characteristic of rifted margin settings. These faults create structural traps that are essential for hydrocarbon accumulation. The extensional tectonics have led to the development of tilted fault blocks, horsts, and grabens, which play a significant role in the distribution of hydrocarbon reservoirs within the region^27,28^.

The evolution of fault trends in the Amal oil field is primarily controlled by the rifting dynamics of the Gulf of Suez. Three main fault trends dominate the area: NW–SE trending faults, which are the dominant rift-related structures; N-S trending faults, which influence the segmentation of reservoirs; and NE-SW trending transfer faults, which accommodate differential extension^12,13,27,29–31^. The interaction of these fault systems has resulted in complex structural configurations that impact reservoir connectivity and fluid migration. The presence of listric faults and rollover anticlines further enhances the trapping potential within the Amal field, making it a geologically favorable site for hydrocarbon exploration^32^.

**Fig. 3:**
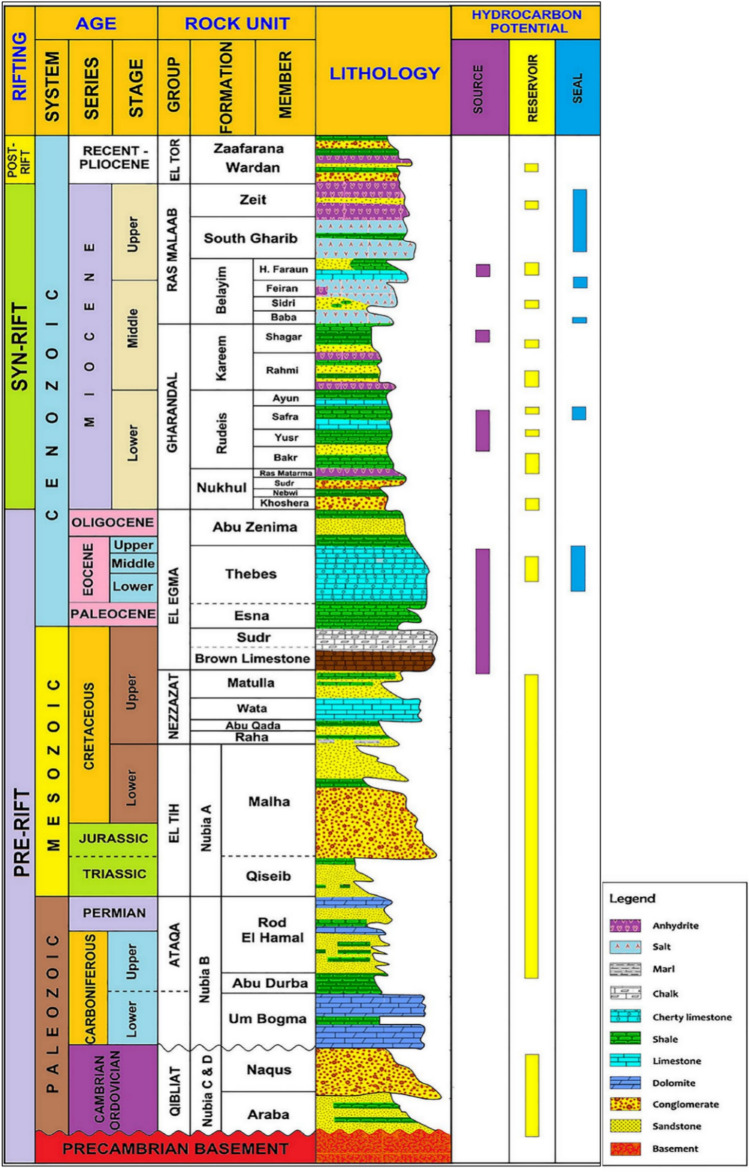
stratigraphic column of the Gulf of Suez’s southern region^41^.

The stratigraphy of the Amal oil field spans from the Precambrian basement rocks to the Quaternary deposits (Fig. 3). The basement is composed of Precambrian granitic and metamorphic rocks, which serve as the structural foundation of the region^33^. Overlying the basement are the Paleozoic to Mesozoic sedimentary sequences, including the Nubian Sandstone, which is an important deep reservoir due to its high porosity and permeability. The Cretaceous succession is represented by carbonate-rich formations such as the Raha and Wata formations. These are followed by the Eocene Thebes Formation, which consists mainly of limestone and is associated with regional unconformities^34,35^.

The Miocene succession, which hosts the primary hydrocarbon reservoirs, is well-developed in the Amal field. It includes the Rudeis, Kareem, Belayim, and South Gharib formations. The Rudeis Formation consists of deep-marine shales and siltstones, serving as a regional seal^30,31^. The Kareem Formation is characterized by alternating sandstone, shale, and carbonate sequences, making it a productive reservoir. The Belayim Formation contains deltaic sandstones and shales, providing excellent reservoir quality^36^. The South Gharib Formation consists of evaporites, which act as cap rock, effectively sealing the underlying reservoirs and preventing hydrocarbon leakage. Above the Miocene formations, the Pliocene and Quaternary deposits are composed of unconsolidated sands and silts^37^.

The Amal oil field’s geology is particularly well-suited for Carbon Capture and Storage (CCS) due to its unique structural and stratigraphic characteristics. Located within the Gulf of Suez rift system, the field features a series of normal faults and tilted fault blocks that create effective structural traps for CO_2_ storage. The primary hydrocarbon reservoirs, including the Miocene-aged Rudies, Kareem, and Belayim formations, exhibit excellent porosity and permeability, making them ideal candidates for CO_2_ injection and long-term storage. Additionally, the presence of robust sealing units, such as the South Gharib evaporites and deep-marine shales, ensures the containment of injected CO_2_, preventing leakage^38^. The field’s mature infrastructure and history of hydrocarbon production further enhance its suitability for CCS, offering opportunities for enhanced oil recovery (EOR) through CO_2_ injection. These geological features position the Amal oil field as a promising site for CCS initiatives, supporting Egypt’s efforts in sustainable energy practices and emissions reduction^39^.

## Material and methodology

**Fig. 4:**
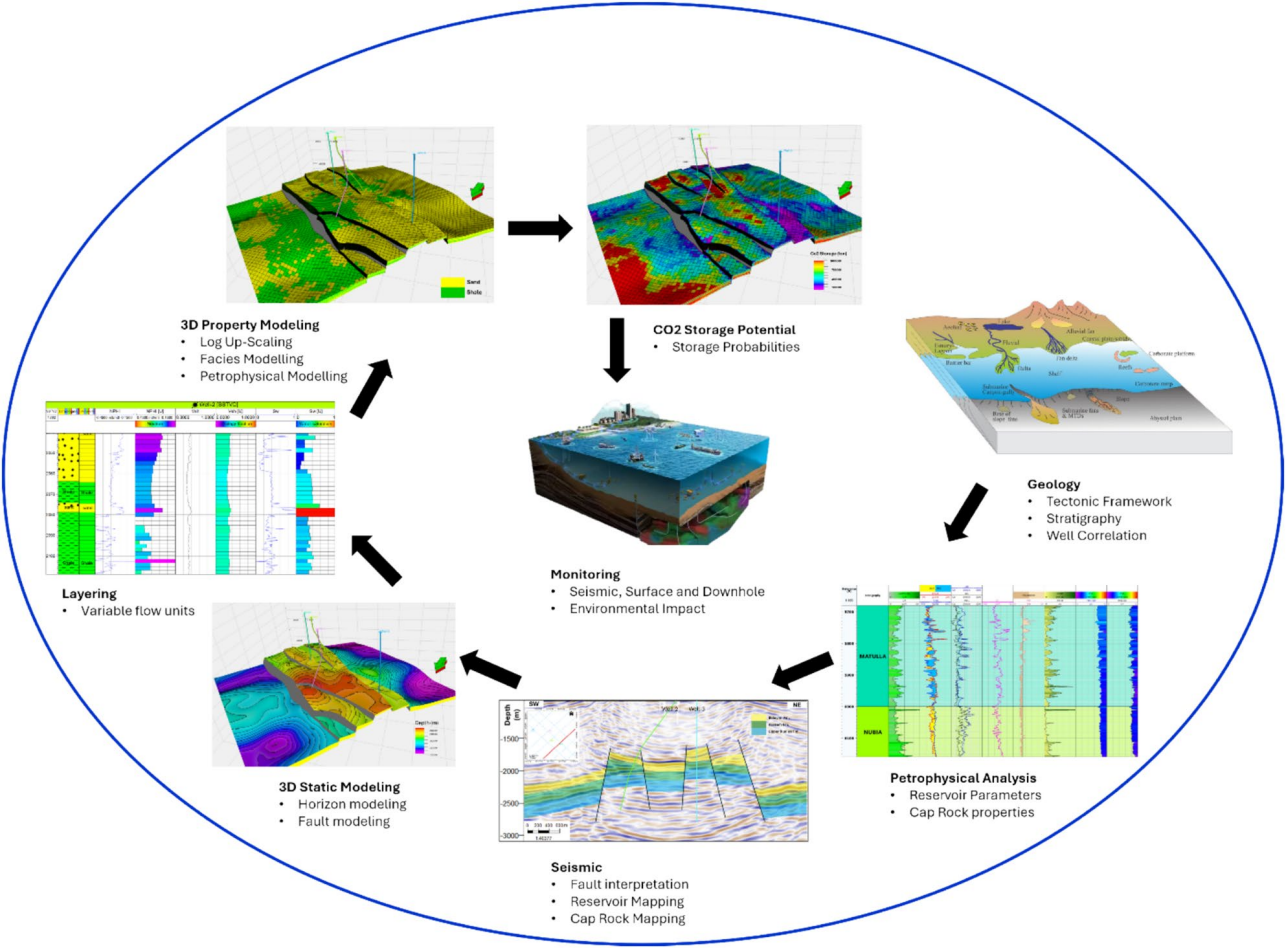
Carbon Capture and Storage Assessment Workflow.

The assessment of Carbon Capture and Storage (CCS) follows a systematic workflow (Fig. 4) that integrates geological, geophysical, and reservoir engineering techniques to evaluate the feasibility and effectiveness of CO_2_ sequestration. Structural interpretation involves fault mapping and horizon picking, ensuring a comprehensive geological framework that accounts for potential migration pathways and reservoir compartmentalization^35^. Advanced geostatistical methods, such as SGS algorithms, are employed to refine spatial variability in reservoir properties, thereby improving the accuracy of CO_2_ storage capacity predictions^42^.

Once the geological framework is established, the CCS assessment advances to risk evaluation, where CO_2_ trapping mechanisms, and long-term containment security are analyzed. Key trapping mechanisms include structural, residual (capillary), solubility, and mineralization trapping^3^. The storage capacity is estimated using fundamental equations incorporating parameters such as total pore volume, irreducible water saturation, formation volume factor, and CO_2_ density^39^. In addition, reservoir modeling is conducted to assess the potential for fault reactivation and cap rock integrity under injection-induced pressure changes^28^. The Gulf of Suez region benefits from thick and strong cap rocks, particularly the Zeit and South Gharib formations, which have the capacity to sustain very high pressures, ensuring long-term CO_2_ containment and minimizing leakage risks. Risk assessment methodologies, including the bowtie method and fault stability analysis, help identify and mitigate potential leakage pathways^43^. To ensure long-term storage security, monitoring and verification strategies are implemented, incorporating time-lapse seismic surveys, microseismic monitoring, and pressure surveillance to track CO_2_ plume evolution and detect anomalies^4,44^. This comprehensive workflow, integrating geological characterization, dynamic modeling, and risk assessment, ensures the safe and effective deployment of CCS technologies, supporting global efforts to mitigate greenhouse gas emissions and transition to a sustainable energy future.

### Dataset

The study utilized 2D seismic lines in the depth domain, stored in SEG-Y format, along with in-lines, crosslines, and wireline log data from four wells. These logs included measurements of Gamma Ray (GR), Density, Neutron, Sonic, and Resistivity. The 2D seismic data, together with the wireline log information, were integrated into Petrel software to construct 3D structural and property models. Additionally, petrophysical analysis was carried out using Techlog software.

The use of 2D seismic data in geological studies presents several limitations, including lower resolution and limited spatial coverage compared to 3D seismic data. These limitations can hinder the accurate characterization of subsurface structures and reservoir properties, which are crucial for effective geological analysis. Additionally, 2D seismic data often suffers from issues such as insufficient offsets, shallow target depths, and data degradation over time. To address these challenges, the data was integrated with surface geology, well data, and nearby studies to enhance the accuracy of subsurface models. Advanced techniques, such as SGS algorithms, were employed to further improve the reliability of geological interpretations. These efforts have significantly increased the confidence in geological assessments derived from 2D seismic data.

### Methodology

All available datasets were integrated to develop 3D structural models for the Upper Rudies formation. Following the construction of the 3D static models, various simulation techniques were applied to model and populate key petrophysical parameters (such as shale volume, total porosity, effective porosity, water saturation, and permeability) within the static model. These models were then utilized and integrated to estimate and evaluate the CO_2_ storage potential.

#### 3D geo-static modeling

This study focused on modeling the Upper Rudies reservoir due to its favorable reservoir properties and facies. The workflow begins with seismic interpretation, starting with the identification and lateral tracking of seismic horizons to map subsurface structures, stratigraphy, and reservoir geometry^12,13^. Additionally, faults and other geological features are mapped to identify subsurface traps, determine their lateral extent, and estimate their volumes^45^. Then 2D structural contour maps were constructed for the desired reservoirs.

The 3D modeling of CO_2_ storage in the study area begins the seismic interpretation outputs, which are the 2D structural surfaces and faults^12,13,46^. These interpreted surfaces are then converted into 3D depth surfaces, allowing for the visualization of structural trends and prospective leads. The next steps include fault modeling, gridding, and horizon generation, where faults provide the foundation for constructing the 3D grid, and interpreted seismic horizons are used to define the vertical layers of the model^47^.

The model incorporates geological surfaces (horizons and faults) and defines the contacts between them, ensuring that the structural model accurately represents the geometry, fault framework, and horizon relationships^48^. To capture essential flow units for accurate CO_2_ storage estimation, the 3D model is subdivided into zones based on the stratigraphy of the study area, with fine layering applied. This detailed structural model, which includes fault systems and reservoir geometry, forms the basis for assessing CO_2_ storage potential. The modeling process ensures the accurate representation of subsurface features, which is crucial for efficient resource utilization^49,50^.

#### 3D property modeling

The property models are generated using output parameters from petrophysical analysis, including facies, shale volume, total porosity, effective porosity, water saturation, and permeability, along with the 3D static model. All estimated parameters are scaled up within the model using arithmetic computation and then populated into the model cells through SGS algorithms.

Facies interpretation was based on the available wireline logs, starting with the discrimination between shale and non-shale zones using Gamma Ray (GR) logs. The sediment types of non-shale zones were then defined using lithology logs, including Neutron, Density, and Sonic logs.

The shale volume (V_sh_) plays a pivotal role in determining reservoir porosity and permeability, directly influencing reservoir quality^51^. To ensure accurate reservoir property estimation, it is essential to compute Vsh correctly. In this study, the Vsh was calculated using the Gamma Ray (GR) method based on Eq. (1). The values obtained from this calculation were then upscaled using arithmetic methods, and a SGS algorithms was applied to spatially distribute the Vsh values across the reservoir model, ensuring accurate representation in areas between wells.

The total porosity is a key property of reservoir rocks, and in this study, the density and neutron logs were used to estimate total porosity, offering a more reliable estimate for these specific formations based on Eq. (2). Effective porosity is a critical factor in permeability estimation. In this study, effective porosity values were calculated using neutron-density porosity data, shale volume and shale porosity as shown in Eq. (3).

The water saturation (Sw) measures the portion of pore space filled with water^52^. To estimate Sw, different models are available, including Archie’s Eq.^53^, which is ideal for clean sand formations, the Indonesian Equation, suited for “dirty” or mixed lithologies, and the Dual Water Model, which adjusts based on lithological variations. Given the presence of thinly interbedded shales in the formations, which can degrade reservoir quality, the Indonesian Equation was applied to calculate Sw. This model, as outlined by^54^, was selected due to its suitability for formations with significant shale content Eq. (4).

Permeability measures the extent to which pore spaces in a rock are interconnected, affecting its ability to transmit fluids. In this study, permeability was estimated to be using established equations due to the lack of core data. The Wyllie-Rose method^55^, a widely used approach in such cases, was applied to calculate permeability, as Eq. (5).

#### CO_2_ storage capacity

The CO_2_ storage potential of a reservoir is evaluated by analyzing its petrophysical properties. Several methods are available to assess CO_2_ storage, including structural trapping, capillary trapping, and solubility trapping. In this study, the CO_2_ storage potential was estimated using petrel software. The estimation process involves utilizing petrophysical data to accurately calculate the capacity for CO_2_ storage within the geological reservoir, as Eq. (6).

1 $$Vsh = \frac{{GR_{log} - GR_{min} }}{{GR_{max} - GR_{min} }}$$

2 $$\emptyset_{T} = \frac{{\emptyset_{N} + \emptyset_{D} }}{2}$$

3 $$\emptyset_{eff} = \emptyset_{T} - \left( { V_{sh} * \emptyset_{sh} } \right)$$

4 $$\frac{1}{{\sqrt {R_{t} } }} = \left[ {\sqrt {\frac{{\varphi^{m} }}{{aR_{w} }}} + \frac{{V_{sh}^{{\left( {\frac{{1 - V_{sh} }}{2}} \right)}} }}{{\sqrt {R_{sh} } }}} \right]S_{w}^{n}$$

5 $$k = a*\frac{{\emptyset_{T}^{b} }}{{Swi^{c} }}$$

6 $$MCO2 = Vpv \times 1 - Swi \times B \times \rho CO2 \times E$$

where Rt represents true resistivity, $V_{sh}$ denotes the clay or shale volume, $GR_{log}$ is the gamma-ray log value, and $GR_{min}$ and $GR_{max}$ are the minimum and maximum gamma-ray values, respectively. $\emptyset_{T}$ refers to total porosity, $\emptyset_{N}$ is neutron porosity, $\emptyset_{D}$ stands for density porosity, $\emptyset_{eff}$ is effective porosity, and $\emptyset_{sh}$ represents shale porosity. The tortuosity factor is denoted by " $a$," while $R_{w}$ is the resistivity of formation water, and $R_{sh}$ is the resistivity of shale. $k$ is permeability measured in millidarcies (mD). $\emptyset_{T}$ indicates total porosity, and $Swi$ represents irreducible water saturation. Constants are defined as $a$ = 10,000.0, *b* = 4.5, and *c* = 2. VPV is the total pore volume (m^3^), $B$ is the formation volume factor, ρ CO_2_ is the density of CO_2_, and E is the CO_2_ storage efficiency.

After these computations for the main petrophysical parameters mentioned above, all these parameters are then scaled up within the static model based on the arithmetic computation and then populated inside the cells of the static model based on the SGS algorithms which are significantly control the storage of the CO_2_.

## Results and discussion

### Structural contour maps

**Fig. 5:**
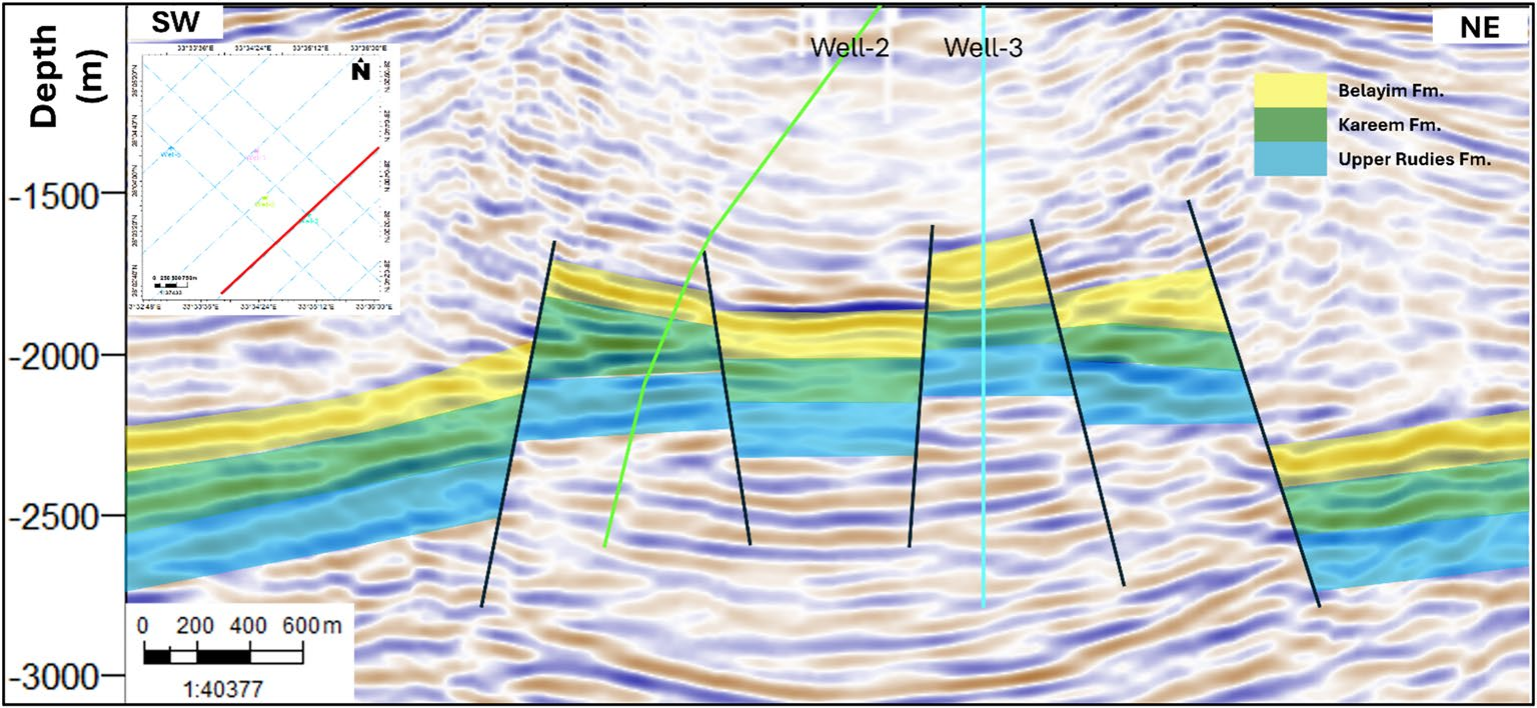
Interpreted Seismic section from southern Amal field showing extensional faults and key horizons.

All available seismic lines were interpreted to track the top and bottom of the Upper Rudies Formation laterally across the area. The horizon is marked by a peak, as the wells transit from sand to shale. Additionally, faults were identified, tracked, and interpreted. Seismic data interpretation reveals that the area is influenced by a series of normal faults, leading to the formation of normal fault structures such as horsts and grabens. The primary structural feature is a horst block, which is bounded by two major normal faults. This block is further affected by minor faults, resulting in the formation of smaller horsts and grabens. These structural features are illustrated in Fig. 5.

**Fig. 6:**
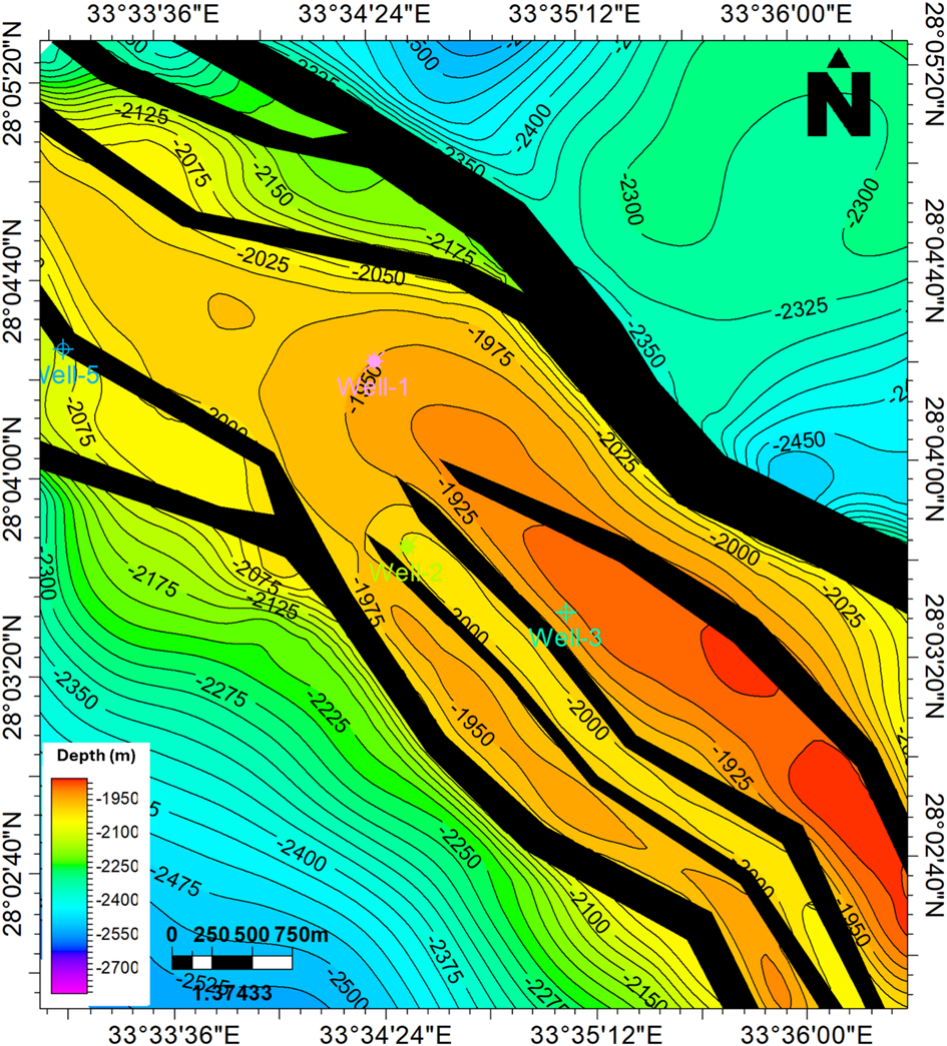
Depth structure contour map of the Upper Rudies Formation with well locations. Black polygons denote fault heaves.

After tracking the Upper Rudies reflector and identifying the associated faults, a 2D structural map was created to analyze the lateral distribution of the present structures across the area. The Upper Rudies map indicates that the region is influenced by a series of extensional normal faults trending NW–SE, following the Clysmic trend. These faults have resulted in the formation of a primary horst block, bounded by two major faults, one with a NE throw and the other with a SW throw. Toward the north, these major faults branch into smaller faults, while in the south, the main horst block is further affected by minor faults, leading to the formation of smaller horsts and grabens. The depth of the Upper Rudies Formation ranges from 1900 to 2800 m. These structural features create a prolific trap suitable for CO_2_ CO_2_ storage, as illustrated in Fig. 6.

### 3D geo-static model

The assessment of CO_2_ storage capacity in the Amal Field requires a comprehensive three-dimensional (3D) structural modeling approach to accurately analyze the present subsurface structures. This process enables detailed inspection of the subsurface by generating variable cross-sections of the constructed model in any direction, allowing for a more precise evaluation of the heterogeneous reservoirs^12,13^. The structural modeling process was carried out in multiple phases, each building upon the outputs of seismic interpretation, including well tops, thickness maps, interpreted horizons, and identified faults. The first phase involved defining the fundamental structural geometry based on seismic data to ensure an accurate representation of subsurface features. Next, a fault framework was constructed using the interpreted faults, establishing the structural framework of the reservoir. Finally, the Upper Rudies horizon was modeled with a detailed definition of the relationships between faults and their structural influence on the reservoir. By integrating these phases, the 3D structural model provides a more precise and dynamic representation of the subsurface, enhancing the assessment of CO_2_ storage potential and ensuring a better understanding of reservoir behavior.

**Fig. 7:**
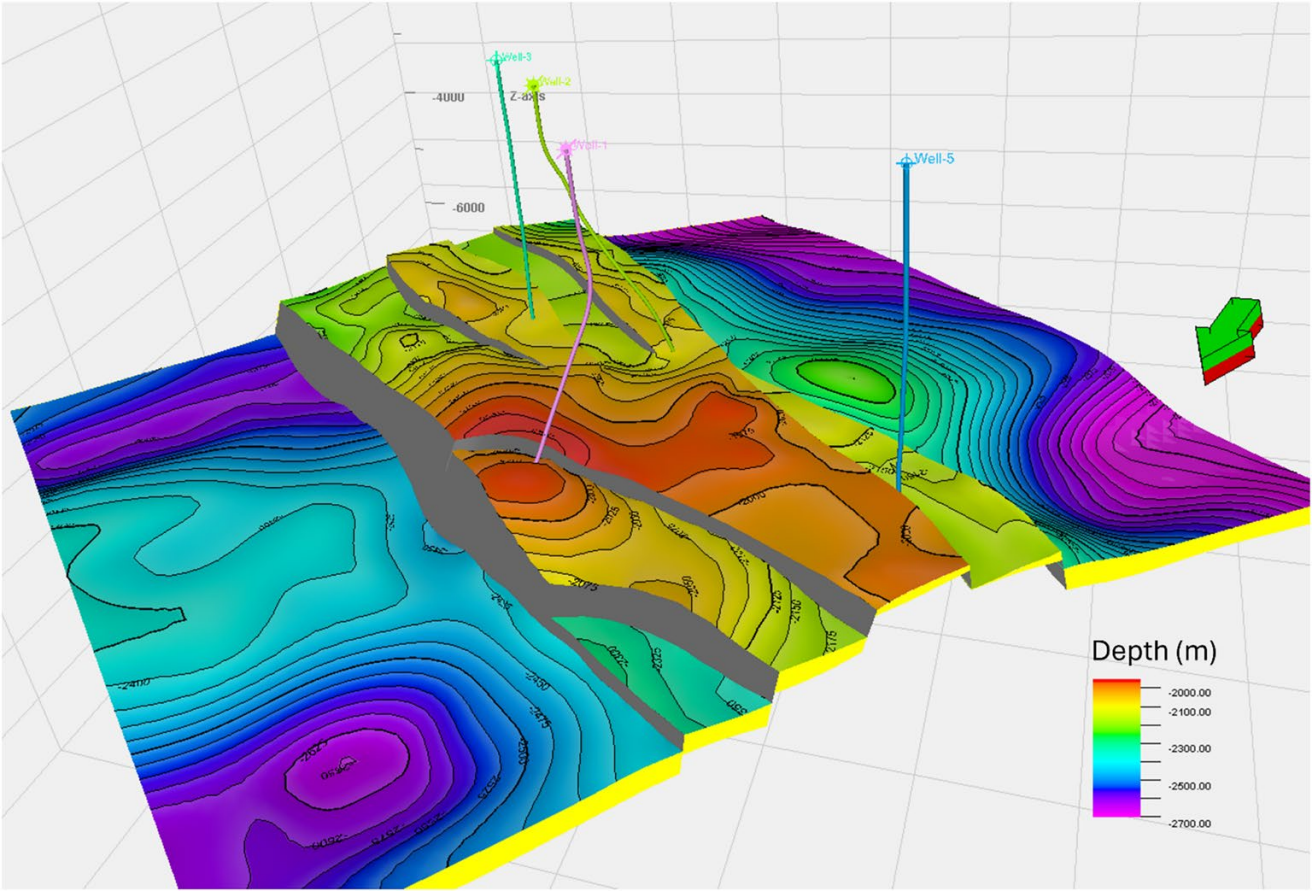
Three-dimensional structural model of the study area, with delineated Upper Rudies zone.

The Upper Rudies model reveals a primary horst block bounded by two major normal faults trending NW–SE, with one fault throwing NE and the other SW, exhibiting significant displacement. Toward the north, these major faults branch into smaller faults, while in the south, the main block is affected by minor faults, resulting in the formation of small-scale horst and graben (Fig. 7). These structural features create an ideal trap for CO_2_ storage. Additionally, the constructed model indicates that the thickness of the Upper Rudies Formation increases toward the south.

Following the construction of the static model, it was essential to capture the reservoir’s favorable properties. To achieve this, the Upper Rudies zone was subdivided into 60 layers to accurately track different flow units. The model highlights that the central part, characterized by a structural high corresponding to the horst, is particularly suitable for CO_2_ storage. Moreover, the detailed structural framework, combined with small cell dimensions, enhances the evaluation and assessment of CO_2_ storage potential, ensuring a more precise understanding of reservoir behavior.

### 3D property models

Petrophysical characteristics are fundamental in evaluating the potential for CO_2_ CO_2_ storage within selected reservoir units, as they provide critical insights into the quality, capacity, and overall suitability of the formations. A thorough analysis of key parameters including facies, porosity (ɸ), permeability (k), volume of shale (Vsh), and water saturation (Sw) was conducted to assess reservoir properties and their implications for CO_2_ storage efficiency^27,56–58^.

**Fig. 8:**
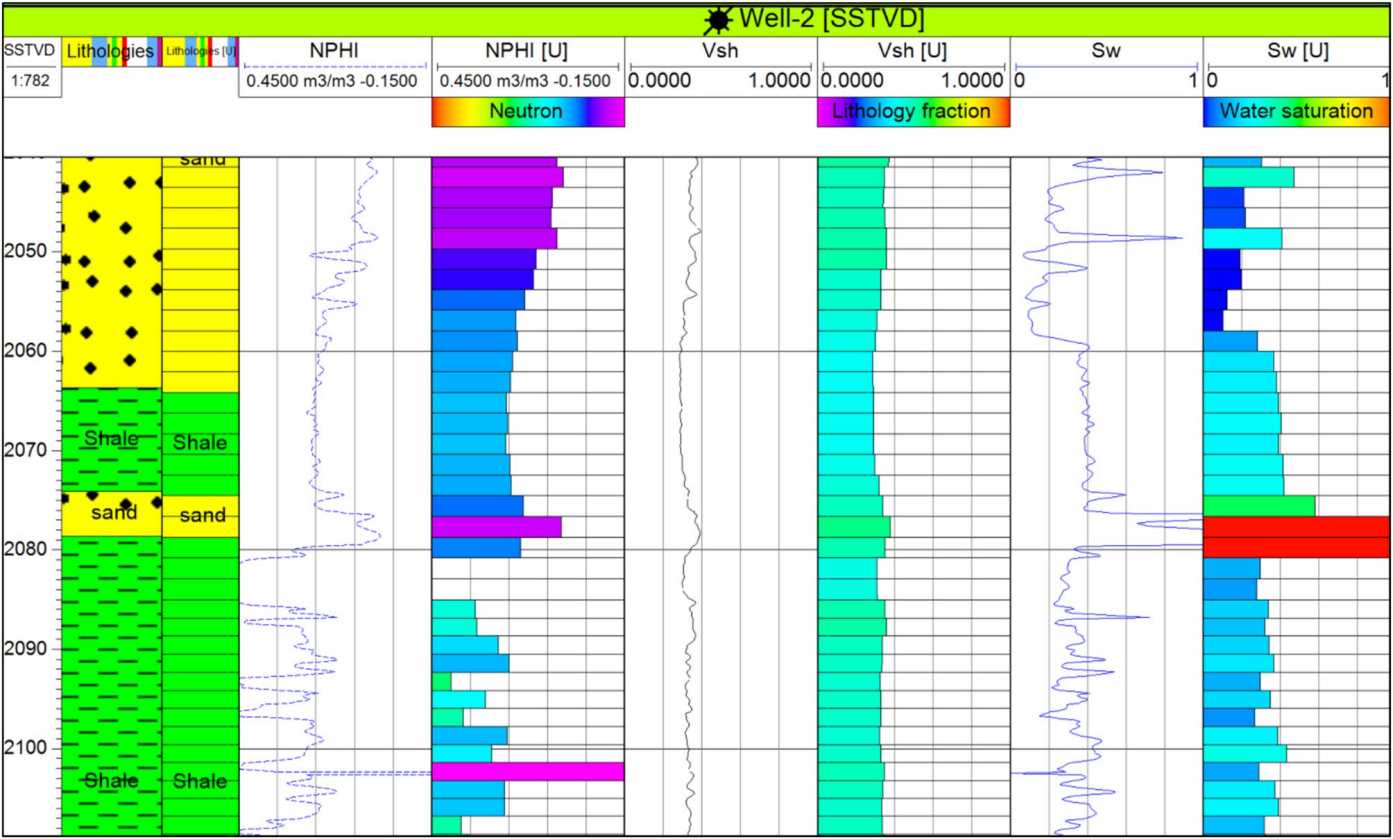
Up scaled well logs of well-2.

The Upper Rudies unit emerged as a particularly promising candidate for CO_2_ storage, as it contains a favorable interval characterized by high porosity and permeability, coupled with low water saturation. These properties enhance the unit’s ability to securely trap and retain injected CO_2_ over the long term. To ensure a detailed and accurate representation of these reservoir properties, well-log data were integrated into the reservoir model and subsequently upscaled (Fig. 8). This process allowed for a more precise modeling of petrophysical characteristics across the formation.

To further refine spatial distribution and enhance resolution, SGS algorithms were applied to propagate these properties across grid cells, improving the model’s predictive capabilities^11,14,15,59^. The resulting petrophysical models, encompassing facies distribution, shale volume, water saturation, effective porosity, and permeability, provided comprehensive insights into the reservoir’s heterogeneity and its capacity for long-term CO_2_ storage. This detailed structural and petrophysical framework is essential for optimizing storage strategies, mitigating potential leakage risks, and ensuring the efficient utilization of the reservoir for CO_2_ sequestration.

**Fig. 9:**
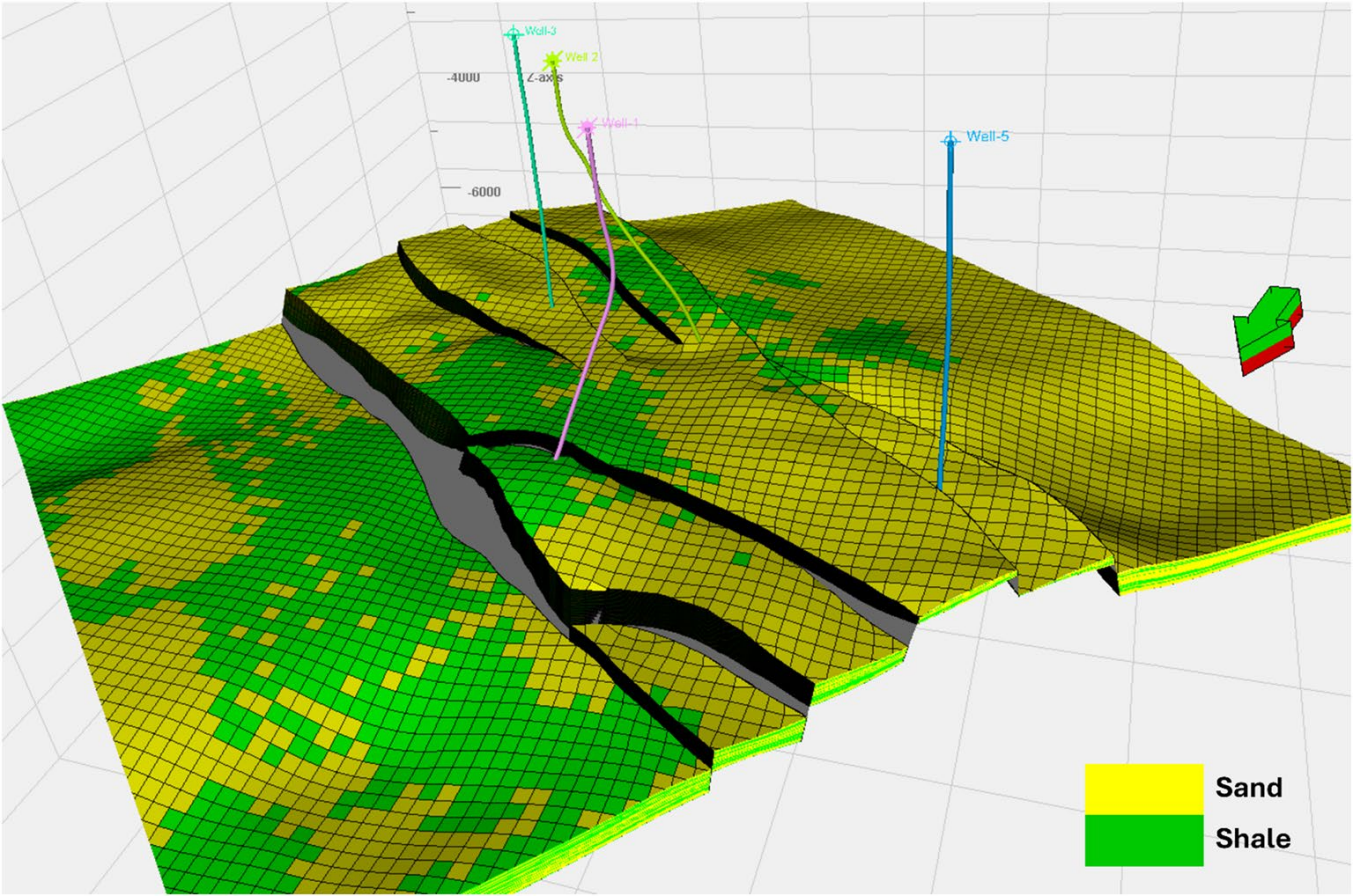
Facies modeling of the Upper Rudies Zone, with sandy facies depicted in yellow and shaly facies in green.

The facies model revealed significant lithological variations within the Upper Rudies zone, with a transition from sand to shale. Lithology was interpreted using available wireline logs, then upscaled to the static model’s cells and distributed throughout the model via the SGS algorithms. The facies model for the Upper Rudies unit indicates a predominance of sandy and shaley facies. Sandy facies are concentrated in the central part of the area, which corresponds to the horst block, and extend into the southern region. In contrast, shale facies are primarily found in the northern part of the area. The model also shows that all wells, except Well-1, encountered sandy facies. Well-1, however, encountered shaly facies (Fig. 9).

Additionally, based on the constructed cross-sections, there are vertical fluctuations in the facies from sand to shale, reflecting the heterogeneity of the formations (Fig. 14a). Despite these variations, the model still identifies a substantial presence of favorable sandy facies that are suitable for CO_2_ storage.

The volume of shale (Vsh) for the Upper Rudies unit was derived from Gamma Ray (GR) logs, upscaled using arithmetic computation, and then distributed across the model with the SGS algorithms. The resulting Vsh model revealed notable variability throughout the unit, reflecting the heterogeneous facies within the study area. Vsh values ranged from 0.05 to 0.65, with higher concentrations found toward the northern and southwestern regions, where shaly patches aligned with the facies model. The shale volume decreases toward the southwest part of the horst but increases moderately toward the northeast direction.

**Fig. 10:**
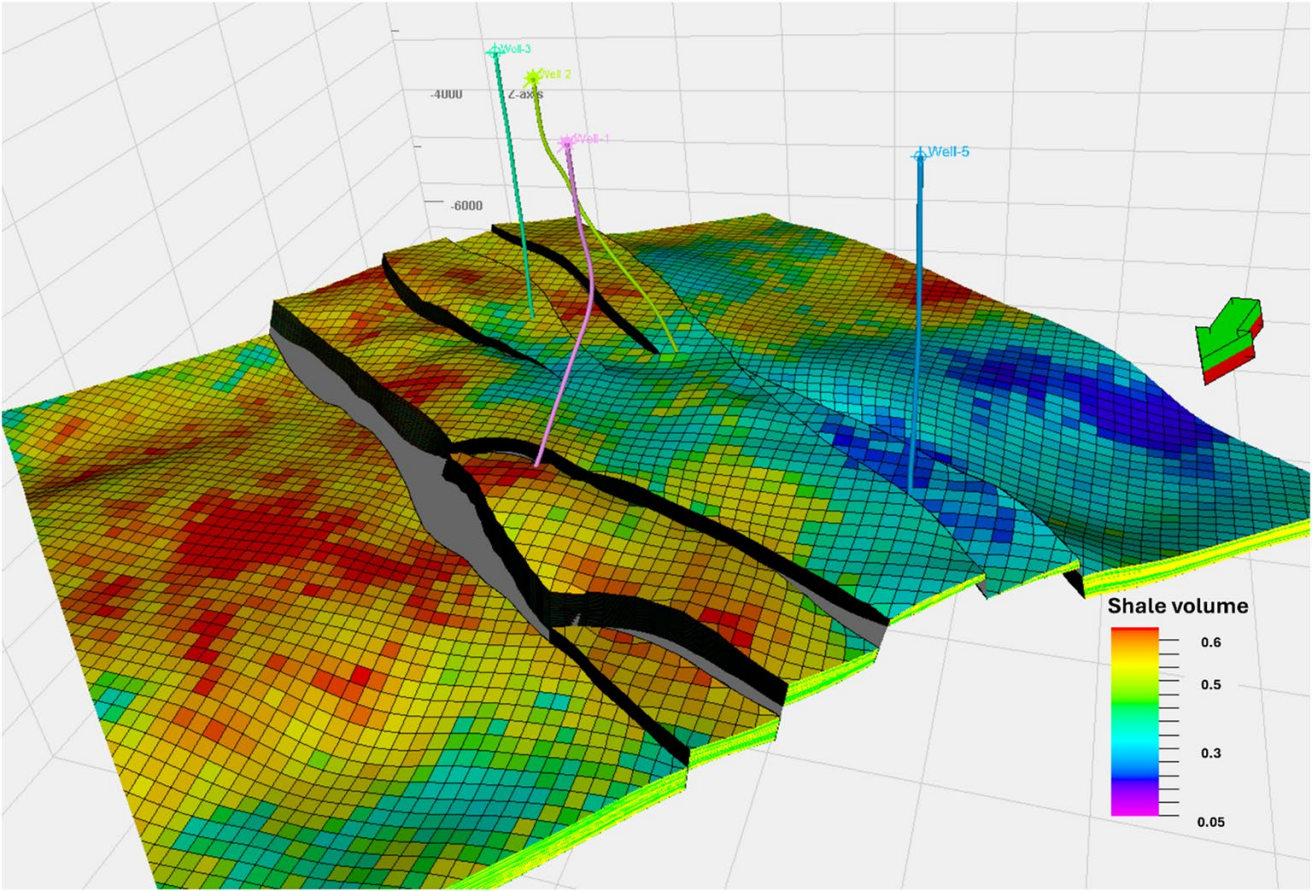
Volume of Shale (Vsh) Model for the Upper Rudies Zone.

These variations are shown in Fig. 10, with structural cross-sections illustrating the Vsh distribution. The model offers a detailed view of shale volume heterogeneity across the reservoir, highlighting fluctuations in Vsh from 0.3 to 0.7 vertically, based on the flow unit. Notably, a wide range of low shale volume exists within this interval, which is favorable for CO_2_ storage (Fig. 14b).

**Fig. 11:**
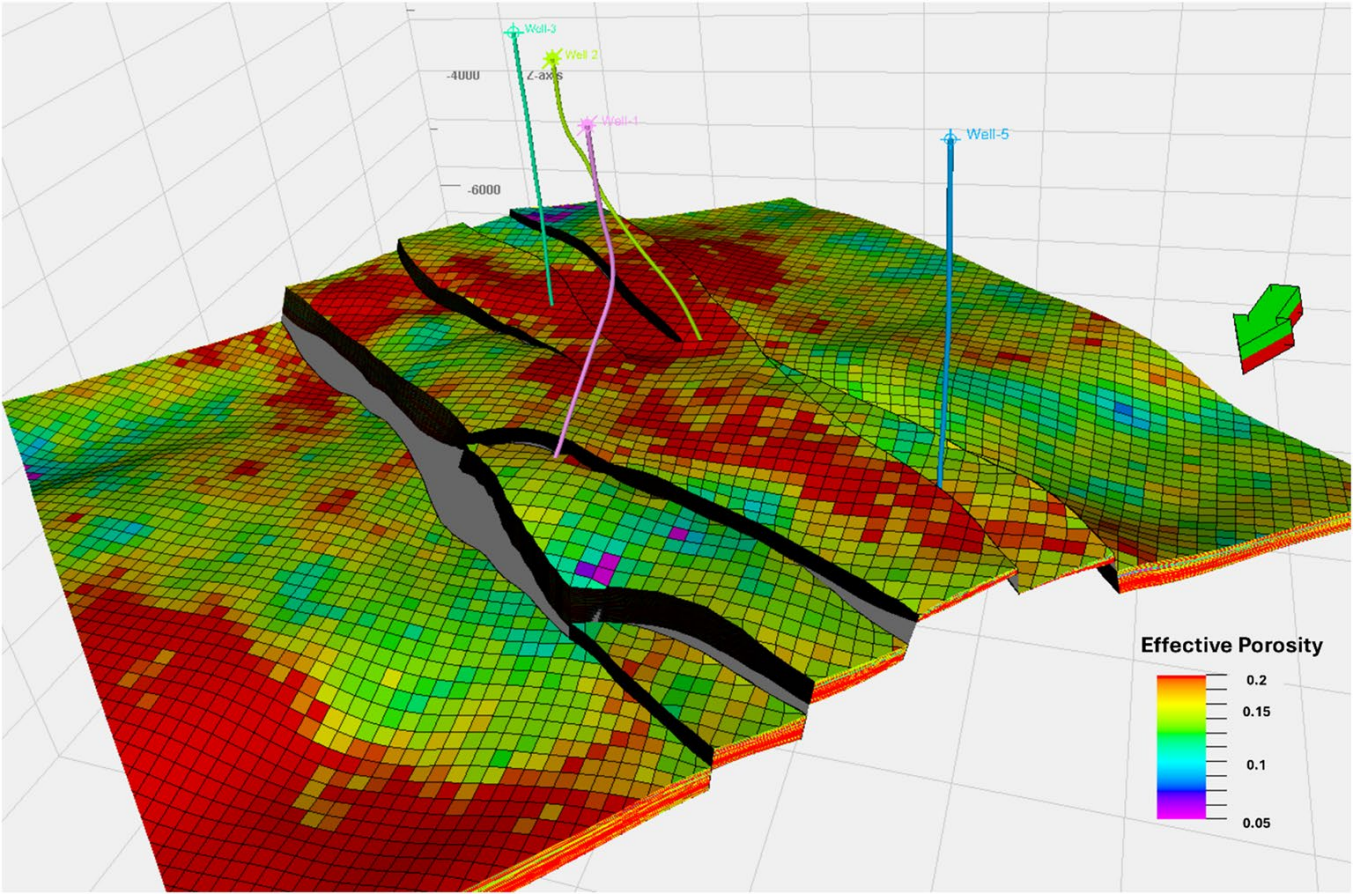
Effective Porosity Model for the Upper Rudies Zone.

The effective porosity model for the Upper Rudies unit was developed using calculated effective porosity values from the available wells (Fig. 11). The model shows uniform lateral porosity values ranging from 0.12 to 0.15, with small regions near the northeast boundary exhibiting lower values of less than 0.12. The horst block displays higher effective porosity values, reaching up to 0.2, although lower values are observed towards the northeastern part of the horst. Well-1 and Well-5 show lower effective porosity values compared to Well-2 and Well-3, which exhibit higher porosity values within the context of the horst block. These results highlight areas with a high proportion of connected pores, offering significant pore space for CO_2_ storage.

Vertically, the effective porosity cross-section (Fig. 14c) reveals heterogeneity in layering, with porosity values ranging from 0.12 to 0.19. These variations further support the potential of the Upper Rudies unit as a viable reservoir. Overall, both the lateral and vertical porosity distributions indicate strong potential for carbon capture and storage (CCS) within the Upper Rudies unit.

**Fig. 12:**
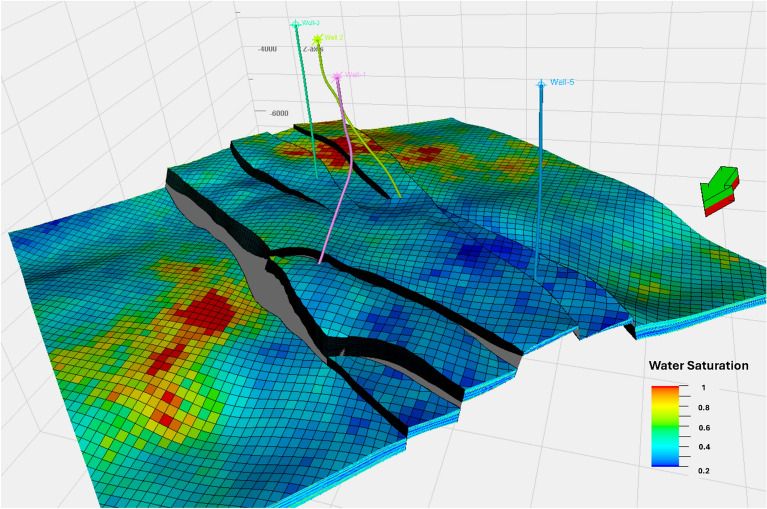
Water Saturation Model for the Upper Rudies Zone.

The water saturation model revealed uniform trend across the study area of lower values in most parts of the model with minor patches of higher values at northern and eastern part of the model with water saturation from 0.8 to 0.95 (Fig. 12). All wells show lower values of water saturation within the horst block. Water saturation values ranged from 0.2 to 0.4 throughout the area, indicating favorable conditions for CO_2_ injection due to the increased availability of pore space. The observed distribution of water saturation aligns with the reservoir’s structural and stratigraphic characteristics, further enhancing its potential for effective CO_2_ trapping through structural, stratigraphic, and residual mechanisms. A vertical cross-section confirmed similar trends, supported by water saturation values derived from the petrophysical analysis of the recorded wells (Fig. 14d).

**Fig. 13:**
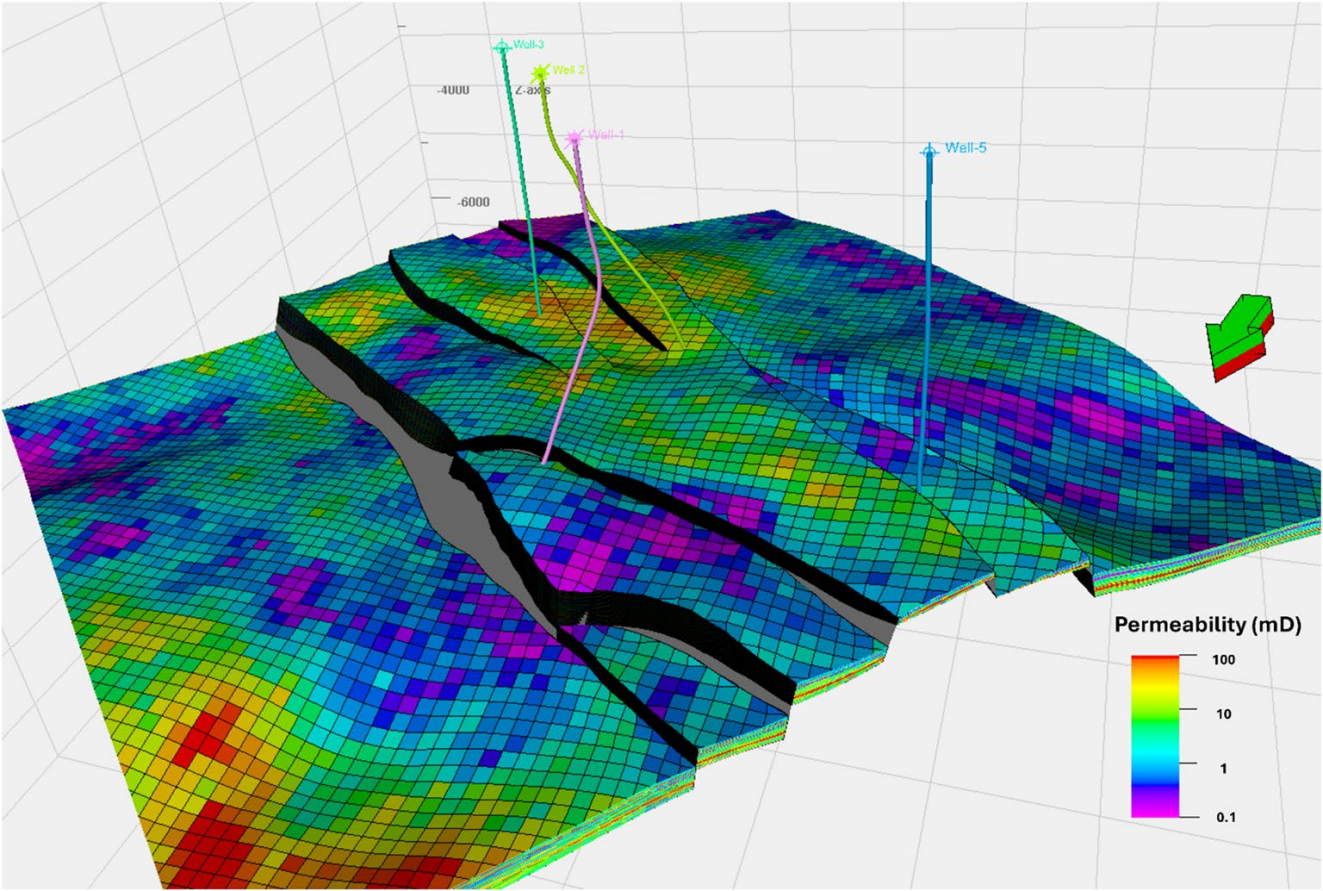
Permeability Model for the Upper Rudies Zone.

**Fig. 14:**
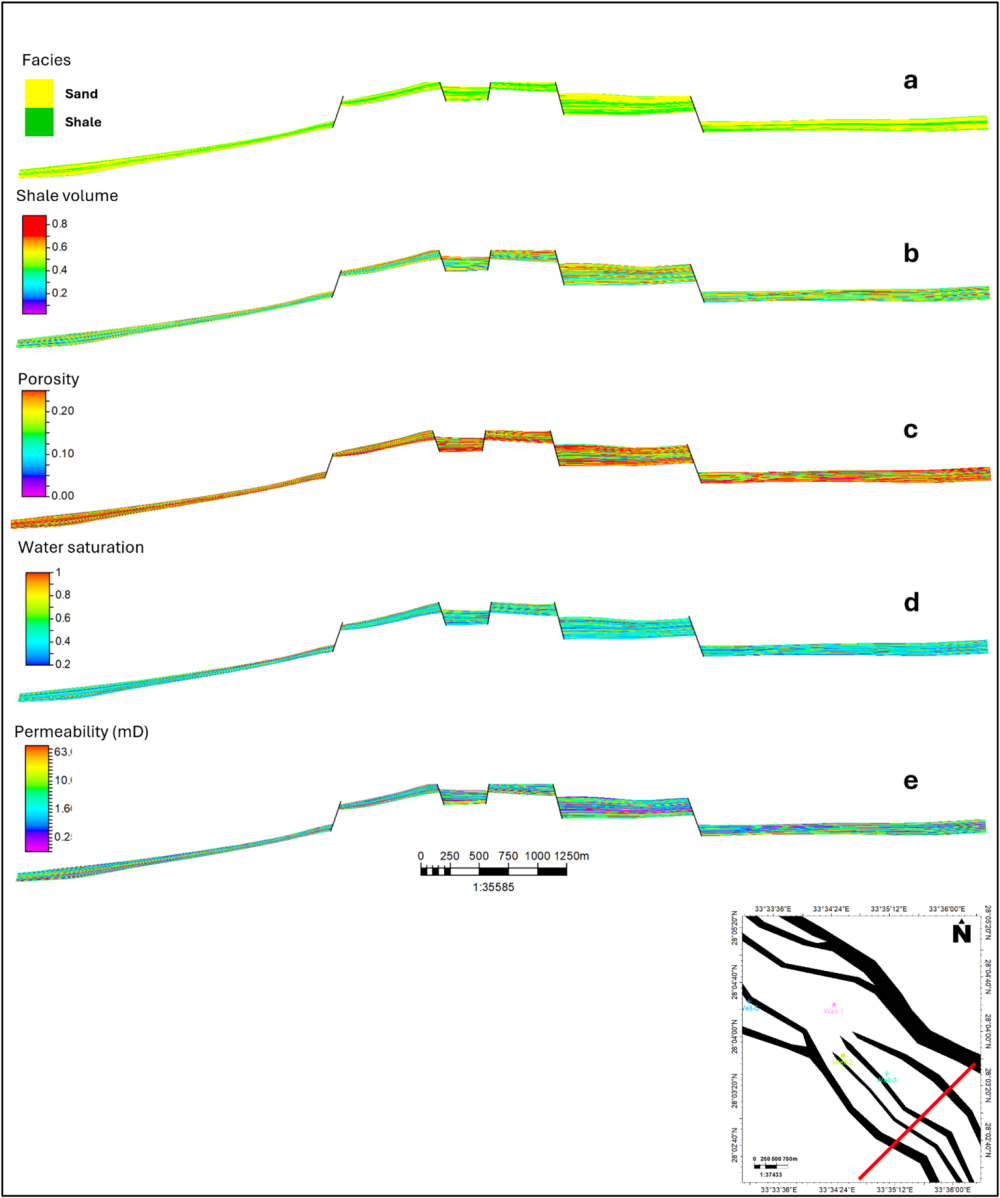
Cross sections illustrating (**a**) Facies, (**b**) Shale Volume, (**c**) Effective Porosity, (**d**) Water Saturation, and (**e**) Permeability of the Upper Rudies Zone.

Permeability modeling within the Amal Field followed a systematic approach, revealing adequate permeability across the Upper Rudies interval (Fig. 13). The permeability model shows lateral variations in values ranging from 0.1 to 100 mD, with semi-uniform values concentrated around 10 mD in the main horst block. There are also vertical variations in permeability, with an average value of 10 mD (Fig. 14e). This permeability is crucial for effective hydrocarbon migration and accumulation, aligning with the structural highs observed in the field. These findings highlight the strong potential of the Upper Rudies as an effective reservoir unit. Its petrophysical properties support the capacity for CO_2_ storage and containment, as demonstrated by the field’s proven reservoir integrity.

### CO_2_ storage potential

**Fig. 15:**
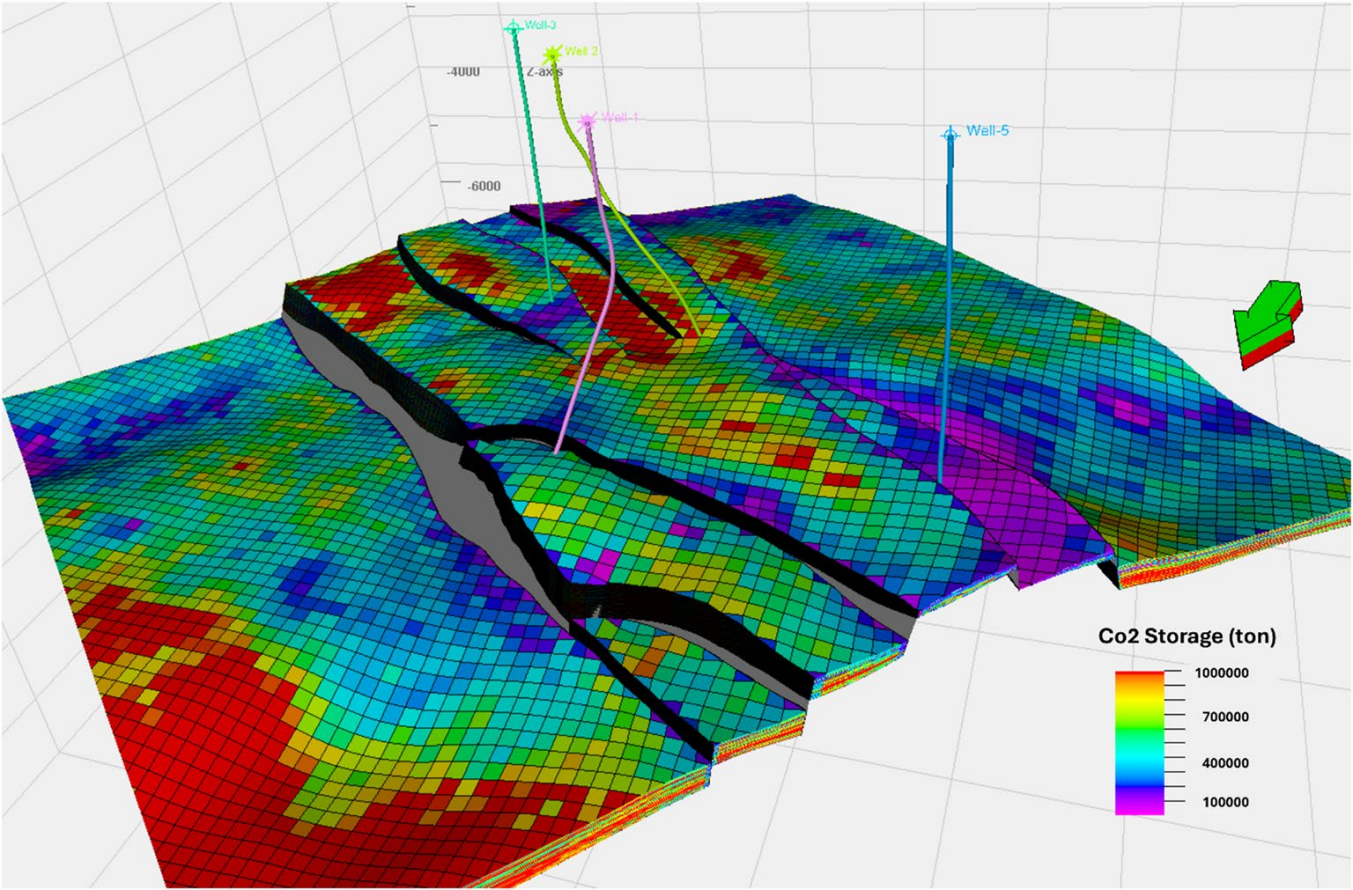
Potential for CO_2_ Storage in the Upper Rudies Zone.

This study assessed the potential for CO_2_ storage (Fig. 15) within a reservoir by combining 3D geological modeling with well log data to derive crucial petrophysical parameters such as net pay thickness, permeability, and porosity. The CO_2_ CO_2_ storage capacity was estimated by considering grid pore volumes, CO_2_ density, formation volume factor, and storage efficiency coefficient. Spatial analysis using Petrel software revealed that the central and northeastern regions of the reservoir have the highest storage potential, primarily due to higher porosity and permeability. In contrast, the southern areas show lower storage potential, attributed to reduced porosity and net pay.

Statistical analysis of the petrophysical properties (P10, P50, P90), as summarized in Table 1, indicates a CO_2_ storage potential ranging from 3.6 to 48.5 million tons, which is favorable compared to CCS sites worldwide, as shown in Table 2^60–65^. While challenges exist in injecting CO_2_ into unconventional reservoirs, the study confirms their suitability for CO_2_ sequestration, with the added benefit of enhancing oil or gas recovery (IOR/IGR) while simultaneously reducing emissions.

**Table 1:** presents the ranking of pore volumes and their corresponding CO_2_ storage potentials, highlighting the relationship between pore volume size and storage capacity.

Probability	Pro volume (× 10^6^ m^3^)	CO_2_ Storage potential (Mt)
P-10	17.7	3.6
P-50	30.3	20.49
P-90	95.8	48.5

**Table 2:** presents a comparison of CO_2_ storage potentials across various sites highlights that Amal field has a strong storage capacity.

CCS Site	Location	CO_2_ Storage potential (Mt)
Quest	Canada	5
In Salah	Algeria	3.8
Petra Nova	USA	1.6
Tomakomai	Japan	0.3

This research fills gaps in understanding CO_2_ storage potential in Egypt’s oil and gas reservoirs and introduces a systematic approach for evaluating storage capacity and addressing uncertainties. The methodology is adaptable to assess other reservoirs in Egypt, contributing to the advancement of CO_2_ geological sequestration (CGS) initiatives and improving strategies to manage data and structural complexities in faulted reservoirs.

### Assessment integrity

The Amal Field primarily produces hydrocarbons from three main Middle Miocene reservoirs: Belayim, Kareem, and Upper Rudies. While most of these reservoirs remain active, the Upper Rudies zone is considered a semi-depleted oil reservoir (DOR). The structural analysis conducted in this study reveals a prolific structural trap in the form of a main horst block, making it a highly viable candidate for CO_2_ storage.

Stratigraphic analysis and well log data indicate that the Kareem Formation, which directly overlies the Upper Rudies, contains a thick shale interval that serves as an effective seal. Additionally, the Zeit and South Gharib Formations, composed primarily of evaporites, act as regional cap rocks, providing further containment for injected CO_2_.

Petrophysical analysis of the available wells demonstrates favorable reservoir characteristics within the Upper Rudies. The shale volume is low, effective porosity is moderately high, water saturation is low, and permeability is high particularly within the horst block. These attributes collectively confirm the Upper Rudies as a high-quality reservoir suitable for CO_2_ storage. Furthermore, fault displacement in the field has led to juxtaposition, where lateral shale facies act as seals against the sand reservoirs. This sealing mechanism has been validated by the productivity of existing wells.

Given that Upper Rudies currently produces oil at very low rates, CO_2_ injection can be utilized for enhanced oil recovery (EOR), followed by long-term CO_2_ storage. Over time, as other reservoirs in the field approach depletion, they may also serve as potential CO_2_ storage sites, given their similar structural and stratigraphic characteristics. This study, therefore, provides an analog for future CO_2_ storage applications in these reservoirs.

The Amal Field is equipped with a well-established infrastructure that can be repurposed for CO_2_ storage and sequestration. The field features an extensive network of pipelines, processing facilities, and injection wells originally designed for hydrocarbon production, which can be adapted for CO_2_ transportation, injection, and monitoring. Offshore platforms and onshore processing plants provide essential support for compression and distribution, facilitating large-scale CO_2_ storage projects. Additionally, advanced reservoir monitoring technologies, including seismic surveys and well logging systems, are already in place, ensuring effective tracking of CO_2_ plume movement and long-term containment. The presence of multiple depleted and semi-depleted reservoirs with existing wellbores presents a cost-effective opportunity for CO_2_ injection without the need for extensive new drilling operations. This infrastructure, combined with Egypt’s commitment to carbon capture and storage (CCS) initiatives, positions the Gulf of Suez as a strategic hub for CO_2_ sequestration, supporting regional and global emission reduction goals.

Extensive research is still required, particularly in assessing and monitoring the long-term environmental impacts of CO_2_ storage. This is crucial for evaluating its sustainability, identifying potential risks, and ensuring the effectiveness of mitigation strategies over time.

### Modeling uncertainties

Modeling uncertainties arise due to various challenges such as limited data availability, data quality issues, complexities in reservoir design, and errors during the modeling process. As a result, it is essential to carefully assess the quality, quantity, and complexity of input data across different scales and critically review the assumptions underlying the chosen modeling approach, particularly in the context of static reservoir uncertainty. In this study, we made efforts to achieve accurate interpretations and derive the most appropriate input parameters.

To address data gaps, we incorporated general geological knowledge, local geological data, and relevant prior experience from nearby oilfields. Additionally, several quality control checks were performed to validate the resulting model, including:

- Ensuring there are no negative cells or bulk volumes
- Avoiding non-orthogonal cells, which should always exceed 45 degrees
- Preventing twisted cells, especially near faults
- Comparing the scaled-up logs with the electrical well logs for consistency (see Fig. 8)
- Verifying that the distributed properties align well with the electrical well logs (see Fig. 8)
- Reviewing the distribution of the facilitation to ensure it is reasonable

The primary sources of uncertainty in lithofacies modeling stem from limited data and the quality of geological information. Consequently, there is considerable uncertainty regarding the distribution of facies or flow units in the three-dimensional structural system. To mitigate this, we employed an integrated approach to facies analysis, combining well log interpretation with data from the analyzed wells to reduce the uncertainty in the facies model.

## Conclusion

This study comprehensively evaluates the Amal Oil Field’s potential for Carbon Capture and Storage (CCS) using an integrated approach that incorporates 3D geological modeling, seismic interpretation, and petrophysical analysis. The findings confirm that the field possesses a well-defined structural and stratigraphic framework that is highly suitable for CO_2_ sequestration. The presence of a primary horst block, bounded by major normal faults, creates an effective structural trap, ensuring secure storage for injected CO_2_. Stratigraphic analysis further supports this suitability, with the Kareem Formation acting as a secondary seal, while the overlying Zeit and South Gharib Formations provide additional containment through their thick evaporite sequences.

The petrophysical analysis of the Upper Rudies reservoir reveals favorable reservoir properties, including low shale volume, moderately high effective porosity, low water saturation, and sufficient permeability, particularly within the horst block. These attributes contribute to the reservoir’s ability to retain CO_2_ effectively. The property modeling, conducted through SGS algorithms, highlights the spatial heterogeneity of key reservoir characteristics and confirms that the central horst block exhibits the highest potential for CO_2_ injection. Permeability variations range from 0.1 to 100 mD, with an average of 10 mD in critical zones, supporting the feasibility of large-scale CO_2_ injection and storage.

Storage capacity estimation integrates key parameters such as grid pore volumes, CO_2_ density, formation volume factor, and storage efficiency coefficients. The results indicate that the Amal Field has the capacity to store between 3.6 and 48.5 million tons of CO_2_, with the central and northwestern regions identified as the most promising areas due to their higher porosity and net pay thickness. The study also highlights the potential for enhanced oil recovery (EOR) as an initial stage of CO_2_ utilization before long-term storage.

A key advantage of the Amal Field is its well-developed infrastructure, which can be repurposed for CO_2_ transportation, injection, and monitoring. Existing pipelines, processing facilities, and injection wells originally used for hydrocarbon production can be adapted, reducing the need for significant new investments. Additionally, advanced reservoir monitoring technologies, including seismic surveys and well logging systems, ensure effective tracking of CO_2_ plume movement and long-term containment security. However, the study does not explicitly address certain limitations, such as the reliance on 2D seismic data, which may affect the accuracy of structural interpretations and reservoir characterizations, and data scarcity in some areas, which could limit the robustness of the findings. To address these limitations, future work should include dynamic modeling to better understand CO_2_ plume behavior and reservoir performance over time. Additionally, developing policy frameworks and regulatory guidelines will be crucial to ensure infrastructure readiness and address potential challenges related to CO_2_ transportation, injection, and long-term monitoring.

This study not only provides a comprehensive assessment of the Amal Field’s viability for CCS but also serves as a reference model for similar reservoirs in the Gulf of Suez and beyond. By integrating geological, petrophysical, and engineering data, the research presents a systematic workflow for evaluating CO_2_ storage potential, addressing structural complexities, and minimizing leakage risks. The findings contribute to Egypt’s national carbon reduction initiatives and align with global climate mitigation strategies by promoting the use of depleted hydrocarbon reservoirs for sustainable CO_2_ sequestration.

## Data Availability

The corresponding author has to be contacted in case of any queries or requirement of data.
